# Mandibular osteomyelitis due to *Raoultella* species

**DOI:** 10.1099/jmmcr.0.005140

**Published:** 2018-01-22

**Authors:** Philip W. Lam, Manal Tadros, Ignatius W. Fong

**Affiliations:** ^1^​ Department of Medicine, University of Toronto, Toronto, Ontario, Canada; ^2^​ Department of Medical Microbiology, St. Michael’s Hospital, Toronto, Ontario, Canada; ^3^​ Department of Infectious Diseases, St. Michael’s Hospital, Toronto, Ontario, Canada

**Keywords:** mandibular osteomyelitis, *Raoultella*, trimethoprim/sulfamethoxazole

## Abstract

**Introduction:**

*Raoultella* is a genus of aerobic Gram-negative bacilli belonging to the family *Enterobacteriaceae* that are commonly found in water, soil and aquatic environments. With improved bacterial identification techniques, *Raoultella* species (namely *R. planticola* and *R. ornithinolytica*) have been an increasingly reported cause of infections in humans.

**Case presentation:**

An 85-year-old man presented to hospital with a several-week history of left jaw pain and trismus. His medical history was significant for left mandibular osteomyelitis treated 1 year previously with amoxicillin-clavulanate. On admission, a computed tomography scan demonstrated a 2.6×1.7×1.6 cm peripherally enhancing collection surrounding the left posterior mandibular body. Two aspirates of the abscess grew a bacterium belonging to the genus *Raoultella*, with discordant species identification (*R. ornithinolytica* versus *R. planticola*) using two different techniques. A potential source of infection included a left lower molar tooth which was extracted months preceding the original diagnosis of osteomyelitis.

**Conclusion:**

This is the first case of mandibular osteomyelitis caused by *Raoultella* species reported in the literature. In contrast to other forms of osteomyelitis, the pathogenesis of mandibular osteomyelitis involves contiguous spread from an odontogenic focus. Risk factors for mandibular osteomyelitis include a history of fracture, irradiation, diabetes and steroid therapy. This report adds to the growing literature of infections caused by this genus of bacteria, and raises the possibility of this organism’s role in odontogenic infections.

## Introduction

*Raoultella* is a genus of aerobic Gram-negative bacilli belonging to the family *Enterobacteriaceae* that are commonly found in water, soil and aquatic environments. Formerly grouped under the genus *Klebsiella*, re-classification into its own genus occurred in 2001 following phylogenetic analyses [1]. With improved bacterial identification techniques, *Raoultella* species (namely *R. planticola* and *R. ornithinolytica*) have been increasingly isolated in humans. In this paper, we report the first case of mandibular osteomyelitis caused by *Raoultella* species.

## Case report

An 85-year-old man presented to hospital with a several-week history of left jaw pain and trismus. His medical history was significant for left mandibular osteomyelitis diagnosed approximately 1 year previously, which occurred following extraction of a left lower molar. At the time, he received a 3-month empiric course of oral amoxicillin-clavulanate (875/125 mg twice daily) with resolution of symptoms. Other co-morbidities included emphysema, hypertension and dyslipidaemia. On examination, there was marked induration, erythema and tenderness over the angle of the left mandible. On oral examination, the patient was edentulous with no evidence of fistula.

## Investigations

**Fig. 1.**
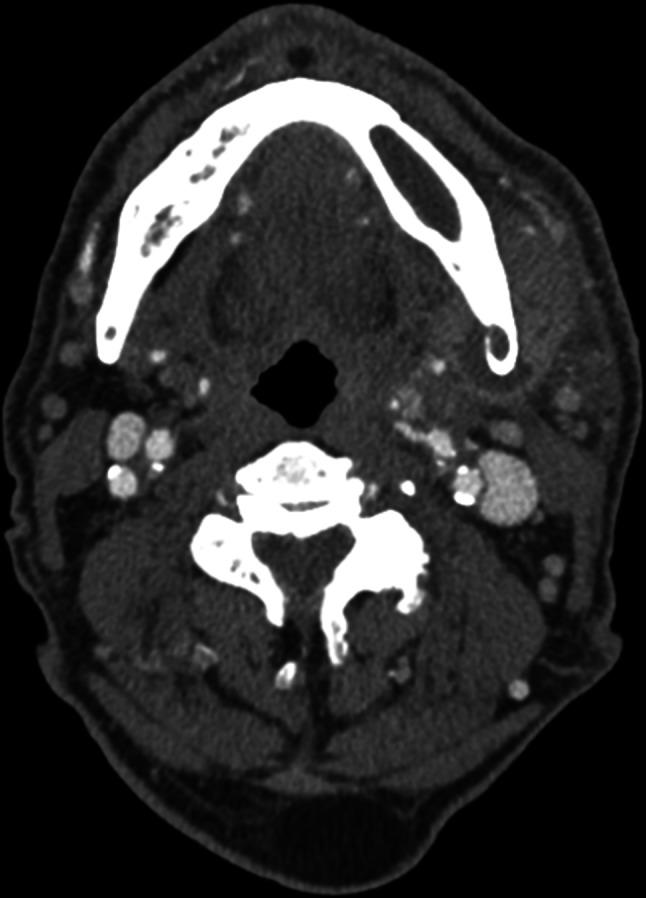
CT image of the neck demonstrating sclerotic lytic lesions of the left mandible, and a 2.6×1.7×1.6 cm hypodense peripherally enhancing collection surrounding the left posterior mandibular body with surrounding oedema.

Laboratory parameters were significant for a white blood cell count of 9.9×10^9^/l (80 % neutrophils), and elevated inflammatory markers (erythrocyte sedimentation rate 32 mm h^−1^ and C-reactive protein 40 mg l^−1^). A computed tomography (CT) scan of the neck demonstrated a new 2.6×1.7×1.6 cm peripherally enhancing collection surrounding the left posterior mandibular body with evidence of chronic osteomyelitis (Fig. 1).

## Diagnosis

A bedside percutaneous aspirate of the abscess was performed, followed by ultrasound-guided drainage by interventional radiology. The specimen was cultured aerobically on Columbia blood agar with 5 % sheep blood, chocolate agar and MacConkey agar No. 3 with Crystal Violet (Oxoid). The specimen was also cultured anaerobically on Brucella vitamin K agar with 5 % sheep blood (Oxoid). Aerobic plates were incubated for 48 h and anaerobic plates were incubated for 4 days. In addition, a broth culture was performed by inoculating the specimen in fastidious anaerobic broth and incubating for 5 days.

Both aspirate samples grew a bacterium belonging to the genus *Raoultella*, with no other organisms recovered. The isolate was negative for ornithine decarboxylase and was identified as *Raoultella planticola* using the VITEK-2 microbial identification system using GNI220 card (bioMérieux) with 99 % probability. The same isolate was identified as *Raoultella ornithinolytica* using matrix-assisted laser desorption ionization time-of-flight (MALDI-TOF) MS (Bruker BioTyper; Bruker Daltonics) with an identification score of 2.47 (Category B). The top nine identification hits were *R. ornithinolytica*, with the 10th hit being *R. planticola*. The isolate was resistant to ampicillin, intermediate to cefazolin and susceptible to ciprofloxacin, trimethorprim/sulfamethoxazole, cefotaxime and piperacillin/tazobactam.

## Treatment

Following the aspiration, the patient received intravenous piperacillin-tazobactam followed by oral amoxicillin-clavulanate (875/125 mg twice daily) 4 days later with gradual improvement in symptoms.

## Outcome and follow-up

The patient’s symptoms resolved completely after 1 month of therapy. He completed an additional 2 months of trimethorprim/sulfamethoxazole (one double-strength tablet twice daily) with normalization of inflammatory markers. Given his clinical response, conservative management (with medical therapy alone) was pursued without any surgical intervention.

## Discussion

With improved bacterial identification techniques, *Raoultella* species (namely *R. planticola* and *R. ornithinolytica*) have been increasingly isolated in humans. Although a significant proportion of these isolates represent colonization, reports of infection have been growing. In a review of 112 cases of *R. ornithinolytica* infection, the most commonly identified sites of infection were the urinary tract, respiratory tract, gastrointestinal tract, skin and wound sites [2]. A similar scope of infections has been reported with *R. planticola* [3]. Risk factors for infection include a history of solid cancer, recent hospital procedure and presence of a catheter [2].

Following a literature review, only one case of *Raoultella* osteomyelitis has been reported [4]. This involved a distal phalanx infected with *R. ornithinolytica* 11 months after surgical fixation of a tendon avulsion [4]. A case of temporomandibular joint infection caused by *R. ornithinolytica* was recently reported in a 38-year-old patient 5 weeks after temporomandibular joint arthroscopy [5].

To our knowledge, this is the first case report of mandibular osteomyelitis caused by *Raoultella* species. In contrast to other forms of osteomyelitis, the pathogenesis of mandibular osteomyelitis involves contiguous spread from an odontogenic focus. Organisms typically implicated in mandibular infections therefore reflect the normal flora and cariogenic organisms that colonize the teeth, and gingival and mucous membranes. Risk factors for mandibular osteomyelitis include a history of fracture, irradiation, diabetes and steroid therapy [6]. Differentiating oral flora from bacteria responsible for infection continues to be a challenge and treatment is often directed against a presumed polymicrobial infection.

Although both *R. planticola* and *R. ornithinolytica* can colonize the upper respiratory and gastrointestinal tract, its role in odontogenic infections is unknown. Its prevalence in these infections may be underappreciated, as many patients with odontogenic infections receive empiric antibiotics without microbiological testing. *Raoultella* species are known to form biofilms [7] and have been isolated from saliva from human subjects [8], but to our knowledge have not been cultured from dental plaque or caries.

Considering the polymicrobial nature of odontogenic infections, it is unusual that both aspirates yielded a monomicrobial result, despite appropriate culturing methods for both aerobic and anaerobic organisms. This may be explained by the administration of several doses of piperacillin-tazobactam prior to aspiration, which may have inhibited growth of other bacteria present at lower concentrations. Due to this possibility, amoxicillin-clavulanate was chosen rather than a more targeted therapy to provide coverage for a potentially polymicrobial infection despite a monomicrobial culture result. The patient’s history of left lower molar tooth extraction months preceding the original diagnosis of osteomyelitis may have served as a portal of entry for infection. Alternatively, the left lower molar may have been infected, with contiguous spread to the underlying bone, but the patient had no recollection of this.

Finally, this report illustrates the ongoing challenges of identifying *Raoultella* isolates to the species level. Discordant species identification was reported by MALDI-TOF MA and VITEK-2. Although uncommon, ornithine-decarboxylase-negative strains of *R. ornithinolytica* have been reported in the literature [9], so this biochemical property is no longer reliable in differentiating *R. planticola* from other *Raoultella* species. Molecular identification techniques were not pursued in our patient as it would not have changed management.

In summary, this is the first case report of mandibular osteomyelitis caused by *Raoultella* species. This report adds to the growing literature of infections caused by this genus of bacteria, and raises the possibility of this organism’s role in odontogenic infections. Further studies are required to better assess the prevalence and risk factors for oral carriage of this organism in the community setting.

## References

[1] Drancourt M, Bollet C, Carta A, Rousselier P (2001). Phylogenetic analyses of *Klebsiella* species delineate *Klebsiella* and *Raoultella* gen. nov., with description of *Raoultella ornithinolytica* comb. nov., *Raoultella terrigena* comb. nov. and *Raoultella planticola* comb. nov. Int J Syst Evol Microbiol.

[2] Seng P, Boushab BM, Romain F, Gouriet F, Bruder N (2016). Emerging role of *Raoultella ornithinolytica* in human infections: a series of cases and review of the literature. Int J Infect Dis.

[3] Ershadi A, Weiss E, Verduzco E, Chia D, Sadigh M (2014). Emerging pathogen: a case and review of *Raoultella planticola*. Infection.

[4] Schmutz N, Adler T, Schelhorn N, Wirz S, Fricker R (2016). Postoperative osteomyelitis of a distal phalanx caused by *Raoultella ornithinolytica*. Handchir Mikrochir Plast Chir.

[5] Levorova J, Machon V, Guha A, Foltan R (2017). Septic arthritis of the temporomandibular joint caused by rare bacteria *Raoultella ornithinolytica*. Int J Oral Maxillofac Surg.

[6] Prasad KC, Prasad SC, Mouli N, Agarwal S (2007). Osteomyelitis in the head and neck. Acta Otolaryngol.

[7] Djeribi R, Bouchloukh W, Jouenne T, Menaa B (2012). Characterization of bacterial biofilms formed on urinary catheters. Am J Infect Control.

[8] Derafshi R, Bazargani A, Ghapanchi J, Izadi Y, Khorshidi H (2017). Isolation and identification of nonoral pathogenic bacteria in the oral cavity of patients with removable dentures. J Int Soc Prev Community Dent.

[9] Walckenaer E, Leflon-Guibout V, Nicolas-Chanoine MH (2008). How to identify *Raoultella* spp. including *R. ornithinolytica* isolates negative for ornithine decarboxylase? The reliability of the chromosomal *bla* gene. J Microbiol Methods.

